# Supplementary material for: Polyamine regulation of ion channel assembly and implications for nicotinic acetylcholine receptor pharmacology
Source: Nat Commun. 2020 Jun 3;11:2799. doi: 10.1038/s41467-020-16629-3 (PMC7271128; doi:10.1038/s41467-020-16629-3)
Supplement: Supplementary file 1 — Supplementary Information [file 41467_2020_16629_MOESM1_ESM.pdf]

Polyamine regulation of ion channel assembly and implications for nicotinic acetylcholine receptor pharmacology

Supplementary Information

Madhurima Dhara, Jose A. Matta, Min Lei, Daniel Knowland, Hong Yu, Shenyan Gu and David S. Bredt

SUPPLEMENTARY FIGURES

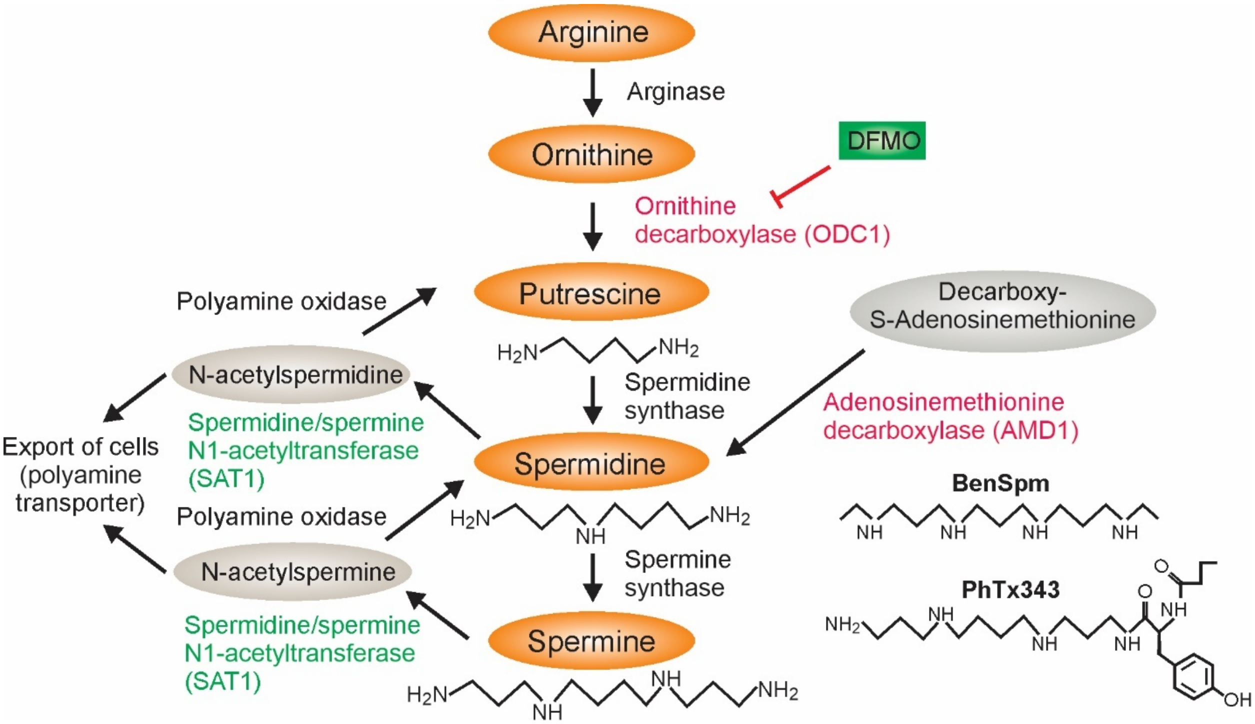

Supplementary Figure 1. Schematic representation of polyamine metabolism

Ornithine decarboxylase (ODC1) is the rate-limiting enzyme for production of polyamines, and spermidine/spermine N1-acetyltransferase (SAT1) is the rate limiting enzyme for polyamine degradation. DFMO inhibits ODC and thereby reduces cellular polyamine levels. The chemical structures for spermidine, spermine and two polyamine analogues (BenSpm and PhTx343) are shown.

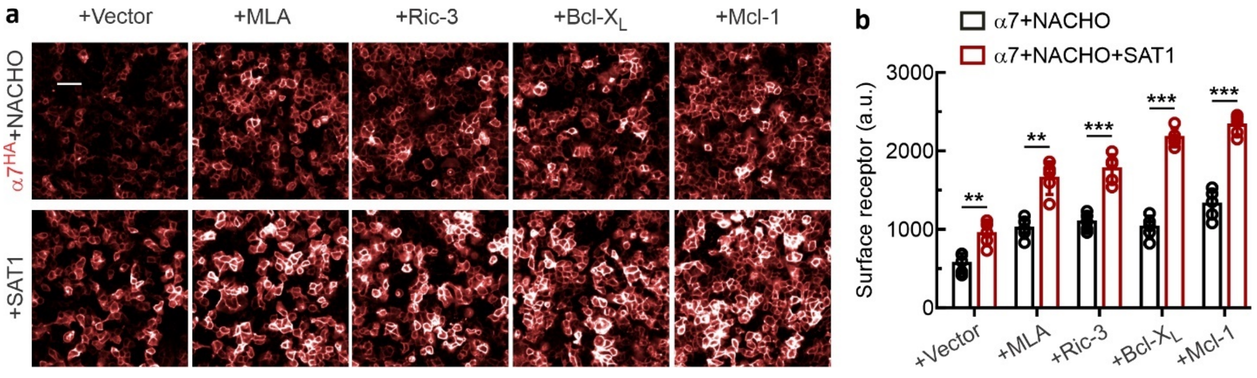

Supplementary Figure 2. SAT1 augments α7 surface expression atop cholinergic ligand, Ric-3, or Bcl-2 proteins

a. Fluorescent anti-HA650 staining of non-permeabilized HEK293T cells transfected with cDNAs encoding HA-tagged α7 and other plasmids or pre-treated overnight with orthosteric ligand MLA (100 μM) as indicated. Scale bar = 50 μm. **b.** Quantification of surface receptors in (a). SAT1 enhanced surface expression of α7 receptor (p=0.007) and also atop MLA (p=0.007), Ric-3 (p=0.0003), and Bcl-2 family proteins (p=0.0002) (n=5). Data displayed as mean ± SD. ** $p < 0.01$, *** $p < 0.001$, Mann Whitney U test versus α7+NACHO. Source data for panel b is provided as a Source Data file.

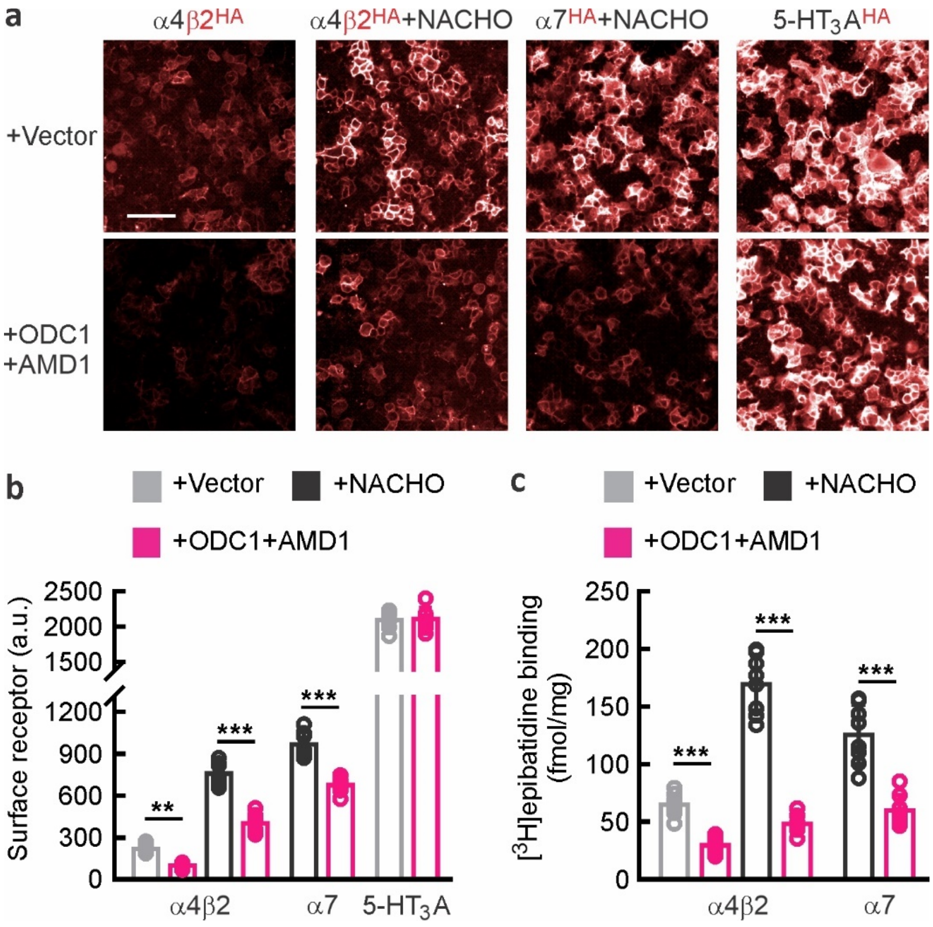

Supplementary Figure 3. Polyamine anabolic enzymes blunt surface expression and assembly of α4β2 and α7 nAChRs

a. HEK293T cells were co-transfected with cDNAs encoding extracellular HA-tagged receptors (α4β2, α4β2+NACHO, α7+NACHO, $5HT_3A$) with or without polyamine anabolic enzymes ODC1 and AMD1. Unpermeabilized cells were labeled with anti-HA650 (Scale bar = 50 μm). **b.** Quantification shows that ODC1 + AMD1 reduced nAChR (α4β2, *p*=0.003 ; α4β2+NACHO, p<$1e^{-4}$; α7+NACHO, *p*=0.0006) but not $5\text{-}HT_3A$ surface expression (*p*=0.9) (*n*=7). **c.** Quantification of $[^3H]$epibatidine binding to cell membranes from HEK293T co-transfected with α4β2 or α7 along with NACHO and ODC1+AMD1 as indicated (*n*=8). ODC1 and AMD1 inhibits $[^3H]$epibatidine binding to nAChRs ($p<1e^{-3}$). Quantifications are displayed as mean ± SD. ** $p < 0.01$, *** $p < 0.001$, One-way ANOVA between the groups for α4β2. Mann Whitney U test versus control for α7 and $5HT_3A$. Source data for panel b and c are provided as a Source Data file.

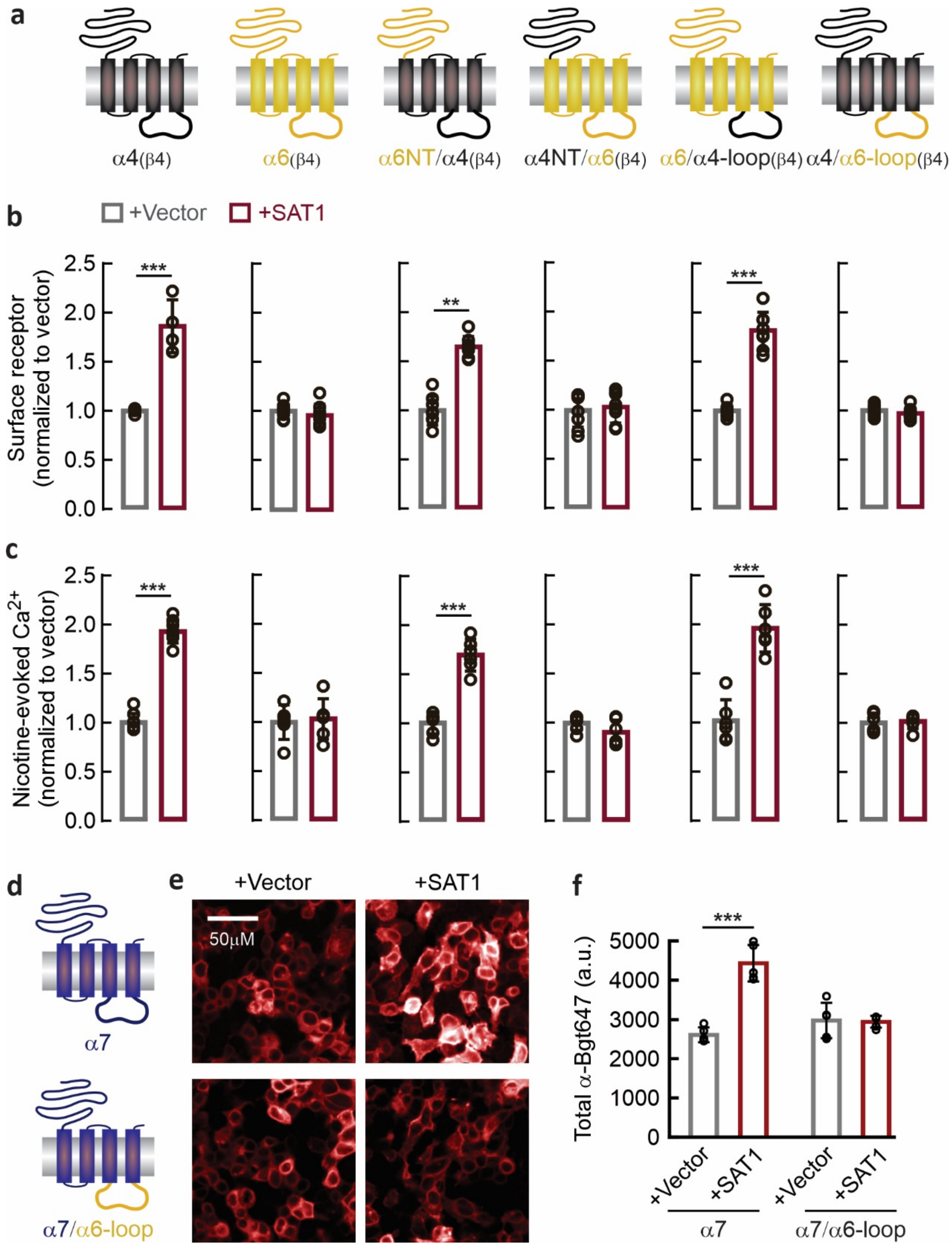

Supplementary Figure 4. *Legend on the following page.*

Supplementary Figure 4. The nAChR TM3-TM4 cytosolic loop determines regulation by SAT1

Graphs (b, c) quantify results presented in Fig. 4. **a.** Schematics of chimeric α4-α6 constructs. **b.** Quantification of fluorescent anti-HA labelling of non-permeabilized HEK293T cells co-transfected with cDNAs encoding α6, α4, α4-α6 chimeric constructs and extracellular HA-tagged β4 with or without SAT1 as indicated. SAT1 enhances surface labelling of α4β4 (p=0.0002), α6NT/α4β4 (p=0.002) and α6/α4-loopβ4 (p=0.0006). All cDNA combinations contained BARP and SULT2B1 (n=6). **c.** Normalized nicotine-stimulated peak Ca^{2+} signal from and wildtype and chimeric α4-α6/β4 receptors co-expressed with SAT1 as indicated (n=6). Similar to receptor surface expression, SAT1 boosts Ca^{2+} signal in α4β4 (p=0.0006), α6NT/α4β4 (p=0.0002) and α6/α4-loopβ4 (p=0.0009). **d.** Schematics of α7-α6 chimeric constructs. **e.** Fluorescent α-Bgt647 staining of permeabilized HEK293T cells transfected with cDNAs encoding α7 + NACHO or α7/α6 loop + NACHO with or without SAT1. **f.** Quantification shows that SAT1 enhanced assembly of the wild type α7 (p=0.0007) but not the α7/α6loop chimeric receptor (p=0.6) (n=5). Data displayed as mean ± SD. ** $p < 0.01$, *** $p < 0.001$, Mann Whitney U test versus receptor + vector for data shown in panels b, c and f. Source data for panel b, c and f are provided as a Source Data file.

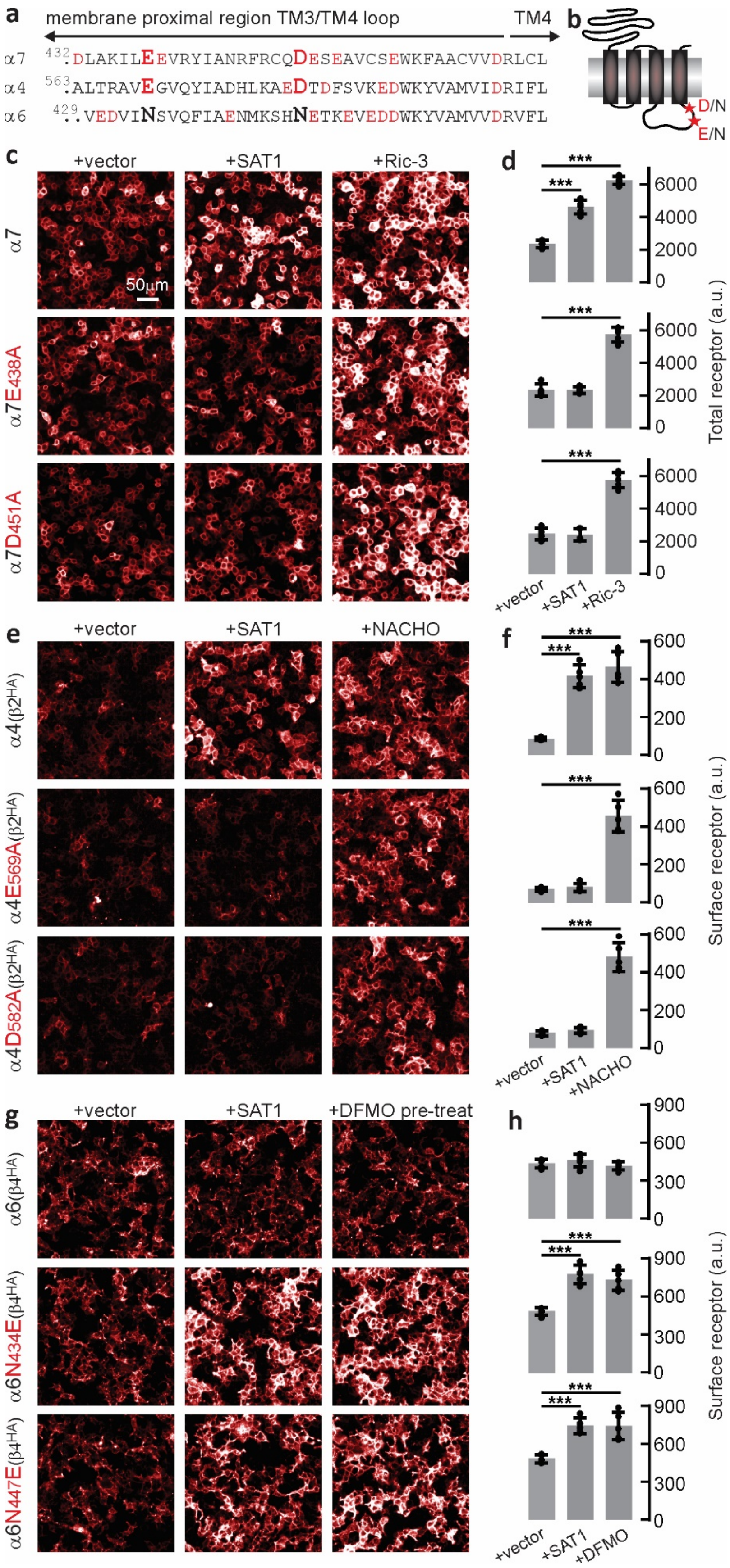

Supplementary Figure 5.
Legend on the following page.

Supplementary Figure 5. Negatively charged residues within the TM3-TM4 cytosolic loop mediate polyamine regulation of nAChR expression

a. Sequences of the TM4 proximal portion of the cytosolic loop aligned for human α7, α4 and α6. Negatively charged residues are shown in red. Residues conserved between α7 and α4 and divergent in α6 are bolded. b. Cartoon of nAChR subunit highlights conserved Glu (E), Asp (D) residues in α7 and α4 that are Asn (N) in α6. **c.** Fluorescent α-Bgt647 staining of permeabilized HEK293T cells transfected with wild type or mutant α7 with or without SAT1 or Ric-3. NACHO was included in all cDNA combinations. **d.** Quantification shows that SAT1 enhanced assembly of the wild type α7 (p<1e^{-4}) but not the mutant receptors (p=0.9), whereas Ric-3 augments assembly for all versions (p<1e^{-4}) (n=5). **e, g**. Fluorescent anti-HA labelling of non-permeabilized HEK293T cells co-transfected with cDNAs encoding α4, α6, or their mutants and extracellular HA-tagged β2 or β4. The cells were also transfected with SAT1 or NACHO or preincubated with DFMO as indicated. For α6β4 receptors, all cDNA combinations contained BARP and SULT2B1. **f.** Quantification shows that SAT1 enhanced surface staining of only the wild type α4β2 (p<1e^{-4}) but NACHO promoted expression of all variants (p<1e^{-4}) (n=5). **h.** Whereas SAT1 co-expression or DFMO preincubation did not affect wild type α6β4 (p=0.2), α6 loop mutants showed modest and significant enhancement (p<1e^{-3}) in surface receptor (n=5). All quantifications are mean ± SD. *** p < 0.001, One-way ANOVA between the groups for panels d, f, h. Source data for panels d, f, h are provided in a Source Data file.

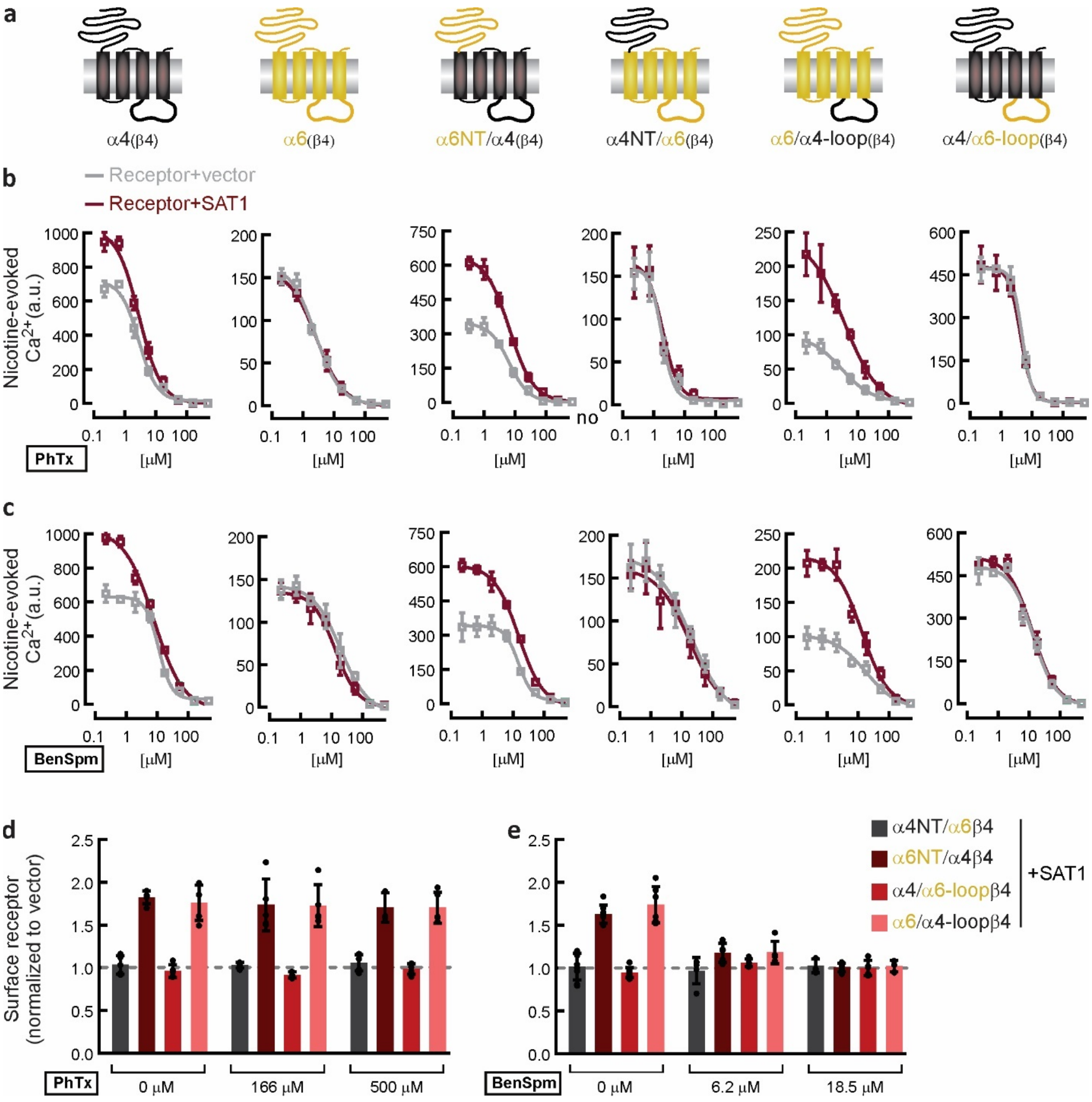

Supplementary Figure 6. *Legend on the following page.*

Supplementary Figure 6. BenSpm but not PhTx-343 blocks SAT1-mediated upregulation of nAChR

a. Chimeric constructs of α4-α6. **(b, c)** Nicotine-evoked Ca^{2+} in HEK293T cells expressing wildtype and α4-α6 chimera/β4-HA receptors transfected with SAT1 as indicted in Fig. 5. PhTx-343 somewhat more potently than BenSpm inhibited nicotine-evoked Ca^{2+} signals for all receptor combinations (*n*=3 for each data point). **(d, e)** Quantification of surface receptor in cells transfected as indicated and treated with PhTx (d) or BenSpm (e). BenSpm but not PhTx-343 blocked SAT1-mediated increase in receptor surface expression. Both SAT1 and BenSpm effects on receptor surface expression required the α4 cytosolic loop (*n*=5). Source data for panel d and e are provided as a Source Data file. Data displayed as mean ± SD.

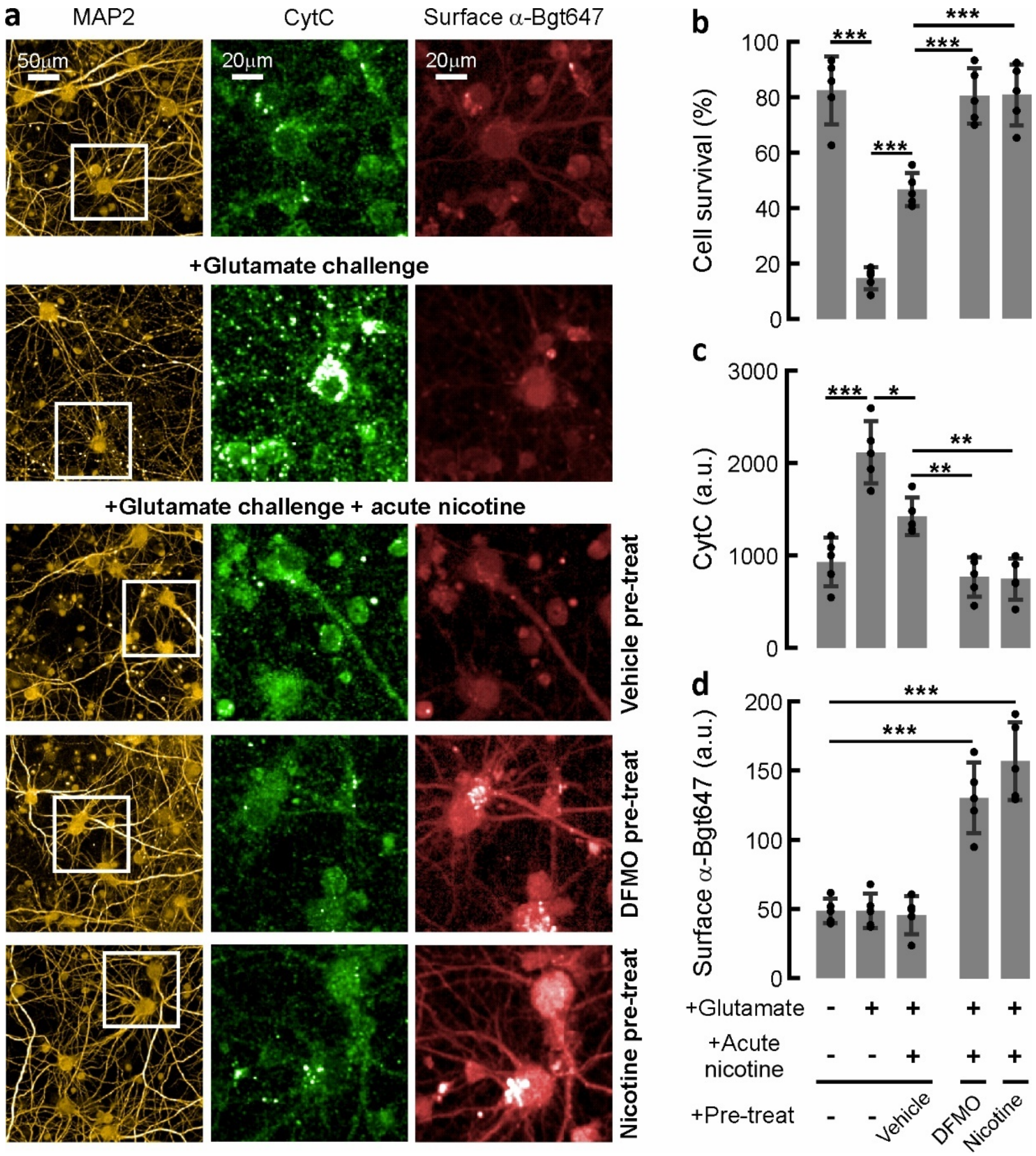

Supplementary Figure 7. *Legend on the following page.*

Supplementary Figure 7. DFMO enhances nicotine-mediated neuroprotection

a. Images of rat cortical neurons (DIV 20) pretreated with DFMO (5 mM) or nicotine (100 μM) and challenged with glutamate (Glu, 30 μM) in the absence or presence of 100 μM nicotine. The cells were stained for MAP2 (left panel) and cytochrome-C (CytC middle panel) and surface α-Bgt647 (right panel). White squares indicate regions that are magnified. **(b-d)** Quantification of percent of surviving cells determined from viable nuclei (see Methods) within the group (b), CytC (c) and surface α-Bgt647 (d) intensity (n=6). Acute nicotine increases cell survival (p=0.0002) and reduces CytC mobilization (p=0.03) during glutamate toxicity. Pre-treatment with either DFMO or nicotine further boosts nicotine-mediated neuroprotection (p<1e^{-4}) and significantly enhances (p<1e^{-4}) surface α-Bgt647. Data displayed as mean ± SD. * $p < 0.05$, ** $p < 0.01$, *** $p < 0.001$, One-way ANOVA between the groups for panels b-d. Source data for panel b-d are provided as a Source Data file.

Supplementary Table 1: List of mutagenesis primers

Protein	Mutation/Chimera	Primers
SAT1	Tyr140Phe	Forward: 5’-TCCATCAACTTCTTCAAAAGGAGAGGTGCTTCTG-3’<br>Reverse: 5’-AAGCACCTCTCCTTTTGAAGAAGTTGATGGATGGTTC-3’
α4 nAChR	Glu247Ala	Forward: 5’-TCCGAGTGTGGTGCAAAGATCACGCTGTGCATC-3’<br>Reverse: 5’-GCACAGCGTGATCTTTGCACCACACTCGGAGGG-3’
	Trp156Ala	Forward: 5’-GAAATTCGGCTCCGCAACCTACGACAAGGCC-3’<br>Reverse: 5’-GGCCTTGTCGTAGGTTGCGGAGCCGAATTTC-3’
	Glu569Ala	Forward: 5’-ACCCGGGCGGTGGCTGGCGTCCAGTACATTG-3’<br>Reverse: 5’-GAAGTCTGTGTCAGCGGCCTTCAGGTGGTC-3’
	Asp582Ala	Forward: 5’- GGCCGAAGCTACAGACTTCTCGGTGAAGGAGG -3’<br>Reverse: 5’- TCTGTAGCTTCGGCCTTCAGGTGGTCTG-3’
α6 nAChR	Asn434Glu	Forward: 5’-GAAGATGTGATTGAGAGTGTTCAGTTCATAG-3’<br>Reverse: 5’-GAACTGAACACTCTCAATCACATCTTCAAC-3’
	Asn447Glu	Forward: 5’-ATGAAGAGCCACGAAGAAACCAAGGAGG-3’<br>Reverse: 5’- CTCCTTGGTTTCTTCGTGGCTCTTCATGTTTTC -3’
α7 nAChR	Glu438Ala	Forward: 5’-GATCCTGGCTGAGGTCCGCTACATTGCC-3’<br>Reverse: 5’-ACCTCAGCCAGGATCTTGGCCAAGTCCG-3’
	Asp451Ala	Forward: 5’-CTGCCAGGCGGAAAGCGAGGCGGTCTGC-3’<br>Reverse: 5’-CTTTCCGCCTGGCAGCGGAAGCGGTTGGC-3’
α6 & α4	α6NT/α4	Forward: 5’-CGAGGTGCGTGCGCCATGCTGACCAGCAAGGGGC-3’<br>Reverse:5’-GGTGTAGAAGAGCGGCAATCTTCTAATGTAGAAAGAATAG-3’
	α4NT/α6	Forward: 5’-CTTGGTACCGCCACCATGGAGCTAGGGGGCCCC-3’<br>Reverse: 5’-CGTGTAAAACATCGGCAGCCGCCGGATGACGAAGG-3’
	α6/α4loop	Forward: 5’- TTTGTGTTGAACATACACCACCGCTCGCCACGC-3’<br>Reverse: 5’- TACCCAAAGAAATACGCGGTCGATGACCATGGCC -3’
	α4/α6loop	Forward: 5’- TTCGTGCTCAACGTGCACTACCGCACCCCAACC-3’<br>Reverse: 5’-CATCCAGAGGAAGATTCTGTCCACCACCATGGCC-3’
α6 & α7	α7/α6loop	Forward:5’-GACGGGGGCAAGATGCCTAGGTGGGTGAAGACAGTTTTCC-3’<br>Reverse:5’-GGCGAACTTCCACTCGCTTTCTACCTCCTTGGTTTCATTGTGG-3’
