# Supplementary material for: Polyamine regulation of ion channel assembly and implications for nicotinic acetylcholine receptor pharmacology
Source: Nat Commun. 2020 Jun 3;11:2799. doi: 10.1038/s41467-020-16629-3 (PMC7271128; doi:10.1038/s41467-020-16629-3)
Supplement: Supplementary file 3 — Reporting Summary [file 41467_2020_16629_MOESM3_ESM.pdf]

nature research

Corresponding author(s): David S. Bredt

Last updated by author(s): May 3, 2020

Reporting Summary

Nature Research wishes to improve the reproducibility of the work that we publish. This form provides structure for consistency and transparency in reporting. For further information on Nature Research policies, seeAuthors & Referees and theEditorial Policy Checklist .

Statistics

For all statistical analyses, confirm that the following items are present in the figure legend, table legend, main text, or Methods section.

n/a	Confirmed	
☐	☒	The exact sample size (*n*) for each experimental group/condition, given as a discrete number and unit of measurement
☐	☒	A statement on whether measurements were taken from distinct samples or whether the same sample was measured repeatedly
☐	☒	The statistical test(s) used AND whether they are one- or two-sided<br>*Only common tests should be described solely by name; describe more complex techniques in the Methods section.*
☐	☒	A description of all covariates tested
☐	☒	A description of any assumptions or corrections, such as tests of normality and adjustment for multiple comparisons
☐	☒	A full description of the statistical parameters including central tendency (e.g. means) or other basic estimates (e.g. regression coefficient) AND variation (e.g. standard deviation) or associated estimates of uncertainty (e.g. confidence intervals)
☐	☒	For null hypothesis testing, the test statistic (e.g. *F*, *t*, *r*) with confidence intervals, effect sizes, degrees of freedom and *P* value noted<br>*Give P values as exact values whenever suitable.*
☒	☐	For Bayesian analysis, information on the choice of priors and Markov chain Monte Carlo settings
☒	☐	For hierarchical and complex designs, identification of the appropriate level for tests and full reporting of outcomes
☒	☐	Estimates of effect sizes (e.g. Cohen's *d*, Pearson's *r*), indicating how they were calculated

Our web collection on statistics for biologists contains articles on many of the points above.

Software and code

Policy information about availability of computer code

Data collection	Data were collected using the following software:<br>FLIPR assay: FLIPR Tetra and Screenworks 4.0 (Molecular device).<br>Immunofluorescent assay: Harmony™ (4.9) high-content imaging and Opera Phenix™ (HH14000000) screening instrument (PerkinElmer).<br>Radioligand binding assay: TopCount NXT scintillation counter (PerkinElmer).<br>Electrophysiology: pClamp9 software (Axon Instruments).
Data analysis	Data were analyzed using the following software:<br>FLIPR assay: Screenworks 4.0 (Molecular device).<br>Immunofluorescent assay: Harmony™ and Columbus 2.7 (PerkinElmer).<br>Electrophysiology: pClamp9 software (Axon Instruments).<br>All other data analyses and subsequent significance check were performed using the software GraphPad Prism, Carlsbad, CA.

For manuscripts utilizing custom algorithms or software that are central to the research but not yet described in published literature, software must be made available to editors/reviewers. We strongly encourage code deposition in a community repository (e.g. GitHub). See the Nature Research guidelines for submitting code & software for further information.

Data

Policy information about availability of data

All manuscripts must include a data availability statement. This statement should provide the following information, where applicable:

- Accession codes, unique identifiers, or web links for publicly available datasets
- A list of figures that have associated raw data
- A description of any restrictions on data availability

The source data underlying Figs 1b, d; 2b-d; 3b,c,g,i; 5b,c,e; 6b-e; 7c-e and Supplementary Figs 2b; 3b,c; 4b,c,f; 5d,e; 6b-d are provided in the Source Data File. Detailed data of genomic screening with cDNA clone library and other additional information can be obtained from the authors upon request. Please see author

contributions for specific data sets.

Field-specific reporting

Please select the one below that is the best fit for your research. If you are not sure, read the appropriate sections before making your selection.

- [x] Life sciences
- [ ] Behavioural & social sciences
- [ ] Ecological, evolutionary & environmental sciences

For a reference copy of the document with all sections, see nature.com/documents/nr-reporting-summary-flat.pdf

Life sciences study design

All studies must disclose on these points even when the disclosure is negative.

Sample size	To provide validity of statistical significance between test groups, we used "biological quadruplet or more (n=4 or more)" in each tested condition. Furthermore, we repeated our experiments at least three times to ensure reproducibility.
Data exclusions	No data were excluded from the subsequent analyses.
Replication	All reported data were successfully replicated at least three times.
Randomization	Sample numbers were not randomized as the source samples (e.g. cultured 293T cells or cultured neurons) are same.
Blinding	Most all experimental outcomes (FLIPR, OPERA, Radioligand binding assay) used automated instruments that scored high throughput biological samples using prespecified parameters. This methodology enabled unbiased readouts on large data sets.<br>For electrophysiological experiments, samples preparation and measurements were conducted by separate scientists. This allowed unbiased data collection.

Reporting for specific materials, systems and methods

We require information from authors about some types of materials, experimental systems and methods used in many studies. Here, indicate whether each material, system or method listed is relevant to your study. If you are not sure if a list item applies to your research, read the appropriate section before selecting a response.

Materials & experimental systems

n/a	Involved in the study	
[ ]	[x]	Antibodies
[ ]	[x]	Eukaryotic cell lines
[x]	[ ]	Palaeontology
[ ]	[x]	Animals and other organisms
[x]	[ ]	Human research participants
[x]	[ ]	Clinical data

Methods

n/a	Involved in the study	
[x]	[ ]	ChIP-seq
[x]	[ ]	Flow cytometry
[x]	[ ]	MRI-based neuroimaging

Antibodies

Antibodies used	HA (Mouse, Invitrogen™ 2-2.2.14) conjugated to DyLight 650<br>GluA1 N-terminal (Mouse, Millipore-Sigma RH95)<br>Cytochrome C (Mouse, Invitrogen™ 7H8.2C12)<br>MAP2 (Chicken, Millipore-Sigma AB15452)<br>Secondary antibody:<br>Goat anti-mouse IgG (H+L) AlexaFluor 488 (Invitrogen™, A-11001)<br>Goat anti-chicken IgY (H+L) AlexaFluor 555 (Invitrogen™ , A-21437)
Validation	Following antibody validation procedure was followed:<br>HA (Mouse, Invitrogen™ 2-2.2.14) conjugated to DyLight 650: Validated using immunofluorescent analysis of HeLa cells transfected with a construct containing an HA Epitope Tag (manufacturer website). For our experiments, any residual non-specific binding were determined from labeling of untransfected HEK293T and subsequently subtracted from the test group signal.<br>GluA1 N-terminal (Mouse, Millipore-Sigma RH95): Evaluated by Western Blot in rat brain lysate (manufacturer website).<br>Cytochrome C (Mouse, Invitrogen™ 7H8.2C12): Evaluated by Western blot of Cytochrome c expression in HeLa cells (manufacturer website).<br>MAP2 (Chicken, Millipore-Sigma AB15452): Validated for use in IC, IH in mouse tissue and cells (manufacturer website).

Eukaryotic cell lines

Policy information about cell lines

Cell line source(s)	HEK293T (ATCC® CRL-3216™)
Authentication	No formal authentication was done.
Mycoplasma contamination	Cell lines were not tested for mycoplasma contamination.
Commonly misidentified lines (See ICLAC register)	No commonly misidentified cell line was used.

Animals and other organisms

Policy information about studies involving animals; ARRIVE guidelines recommended for reporting animal research

Laboratory animals	E18 Sprague-Dawley Rat (BrainBits).<br>E18 C57BL/6 mice: Wild type and NACHO knockout (Tmem35atm1(KOMP)Vlcg).
Wild animals	The study did not involve wild animals.
Field-collected samples	This study did not use samples collected from the field.
Ethics oversight	All animal experiments reported here were overseen and approved by an AAALAC accredited institutional review board.

Note that full information on the approval of the study protocol must also be provided in the manuscript.
